# Effects of Replacing Corn with an Aged Brown Rice–Wheat Mixture on Laying Performance, Egg Quality and Nutrient Digestibility in Laying Ducks

**DOI:** 10.3390/ani15081088

**Published:** 2025-04-09

**Authors:** Xingyuan Luo, Shiping Bai, Qiufeng Zeng, Xuemei Ding, Jianping Wang, Huanwei Peng, Yan Liu, Yue Xuan, Shanshan Li, Keying Zhang

**Affiliations:** Animal Nutrition Institute, Key Laboratory for Animal Disease-Resistance Nutrition of China Ministry of Education, Sichuan Agricultural University, 211 Huimin Road, Wenjiang District, Chengdu 611130, China; 17745468580@163.com (X.L.); shipingbai@aliyun.com (S.B.); zqf@sicau.edu.cn (Q.Z.); dingxuemei0306@163.com (X.D.); wangjianping1983@hotmail.com (J.W.); phw@sicau.edu.cn (H.P.); liuyan12334@163.com (Y.L.); 71128@sicau.edu.cn (Y.X.); 71449@sicau.edu.cn (S.L.)

**Keywords:** laying duck, aged brown rice–wheat mixture, laying performance, egg quality, alternative feed

## Abstract

This study examined the effects of replacing corn with an aged brown rice (ABR)–wheat mixture (ABR: wheat = 85%:15%) on laying performance, egg quality, yolk fatty acid profile, economic benefits, serum biochemistry, and nutrient digestibility in laying ducks. A 12-week trial with six hundred 32-week-old Jinding ducks were randomly divided into five groups, with 10 replicates per group, and each replicate contained 12 ducks. The levels of ABR–wheat mixture in the diet were 0%, 12.5%, 25%, 37.5%, and 50%, respectively, to replace equal amounts of corn. The basic diet of the control group was corn–soybean meal (corn accounted for 50%). The experimental period was 12 weeks (from 32 to 43 weeks old). No significant differences were observed in laying performances (*p* > 0.05). From a numerical perspective, the duck-housed laying rate and egg mass were highest in the 37.5% group and lowest in the 50% group. Yolk color declined linearly (*p* < 0.05) over 12 weeks. The albumen height and Haugh unit showed a quadratic increase in weeks 4 and 8 (*p* < 0.05). The relative content of monounsaturated fatty acids (MUFA) increased linearly (*p* < 0.05), while the relative content of polyunsaturated fatty acids (PUFA), ω-3 PUFA, ω-6 PUFA, and ω-6/ω-3 PUFA decreased linearly in egg yolks (*p* < 0.05). The triglycerides (TG) content in serum showed a significant secondary change (*p* < 0.05), with the 50% group significantly lower than the other treatment groups (*p* < 0.05). The apparent digestibility of ether extract (EE) in the diets decreased linearly (*p* < 0.05). The apparent digestibility of crude protein (CP) in the diets was significantly reduced with the 50% group (*p* < 0.05), while there was no significant difference between the 0% and 37.5% groups (*p* > 0.05). Compared with the control group, economic analysis revealed net gains of 0.04–0.10 USD/duck at ≤37.5% substitution, but a loss (−0.04 USD) at 50%. In conclusion, the ABR–wheat mixture could reduce the yolk color and yolk PUFA content, but it could improve albumen quality and increase yolk MUFA content. High-level ABR–wheat mixture (50%) significantly reduced the TG content in serum and nutrient digestibility of the diet and also showed a numerical decrease in laying rate and egg mass. Our findings suggest that up to a 37.5% ABR–wheat mixture can effectively replace corn in laying duck diets without negatively affecting laying performance, while improving albumen quality and altering yolk fatty acid composition.

## Simple Summary

Brown rice and wheat are common energy feed substitutes for corn in poultry diets. But there are few reports on the replacement of corn with a mixture of brown rice and wheat. This study aimed to explore the application effect of replacing equal amounts of corn with an aged brown rice–wheat mixture. The results showed that substitution levels of 12.5% to 37.5% did not negatively impact laying performance and economic benefits, but it reduced yolk color and yolk polyunsaturated fatty acids, improved egg white quality, and increased yolk monounsaturated fatty acids. At 50% substitution, nutrient digestibility declined significantly. The highest laying performance and economic benefits could be achieved at a level of 37.5%. Therefore, it is feasible to use a suitable proportion of ABR–wheat mixture as a substitute for corn in egg duck production, which is significant for scientifically destocking aged rice and alleviating corn shortages.

## 1. Introduction

Global corn production is decreasing with climate change [1], while prices continue to rise and fluctuate greatly [2]. Corn, as a primary energy feed ingredient for livestock and poultry, accounts for approximately 50% to 70% of the total energy in poultry diets [3]. With the rapid development of animal husbandry in China, the supply of corn cannot meet the demand [4]. In 2022, 80% of China’s 2.062 billion tonnes of corn imports were used as feed materials [5]. Therefore, it is necessary to effectively promote the reduction and substitution of corn in livestock feed. Rice and wheat are major food crops in China, with extensive planting areas and high yields [6,7]. The inventory of paddy exceeds 100 million tons in China [8]. In major rice- and wheat-producing regions, or during corn price fluctuations, wheat and rice grains are typically less expensive than corn, which can not only help to lower feed costs, but substitute corn in the diet of poultry without impacting performance [9,10]. The aged brown rice produced by dehulling aged paddy is not suitable for human consumption due to its low edible quality after long-term storage [11]. According to national regulations, when the grains, after being stored for a few years, became aged, they would be used as feed mixed with other grains for animal production [12].

The major nutrients in brown rice obtained from paddy hulling are equal to or exceed those found in corn, and when used in livestock feed, brown rice can produce effects on egg production and growth that are comparable to those of corn [13,14,15,16]. Brown rice offers better protein quality and a more balanced amino acid composition [17]. Brown rice’s bran layer includes soluble fiber beneficial for intestinal health and antioxidants such as γ-oryzanol and tocopherol that improve poultry immunity [18]. Wheat has a higher protein content than corn and is rich in methionine [19]. Brown rice and wheat present challenges in practical applications, such as containing anti-nutritional factors that limit animal digestion and absorption [20,21], and having almost no pigments, which diminishes egg yolk color [22]. During storage, the nutritional value of brown rice decreases due to the effects of oxidation, enzymes, microorganisms, and self-respiration [23,24]. Moreover, fungal toxin infection, and oxidative rancidity of fats and proteins, may pose safety risks [25]. As potential feedstuff, aged brown rice or wheat has been used in poultry feed trials, but currently, it is usually one of them that replaces corn, rather than their mixture [26]. He et al. [27] found that, compared to newly harvested brown rice, aged brown rice stored for 6 years reduced the immune function, serum total protein (TP) content, and antioxidant capacity of broilers, but had no significant impact on growth performance, slaughter performance, and meat quality. Yuan [28] observed that replacing 70% of corn with brown rice aged for seven years can lead to increased daily weight gain in meat ducks, but it also lightens their feathers, abdominal fat, subcutaneous fat, and shin color. Adding 0%, 5%, 10%, and 15% brown rice stored for 3 years to the feed of laying hens did not significantly affect egg production performance and egg quality [11]. There are few reports on the use of aged brown rice in laying poultry. However, research has indicated that completely substituting normal corn with aged corn greatly worsens laying hens’ performance, egg quality, and apparent EE utilization, while lowering serum total cholesterol (TC), low-density lipoprotein (LDL-C), and very low-density lipoprotein (VLDL-C) levels [29], and reducing PUFA in egg yolk [30]. Moreover, adding 15% to 45% wheat to the diet for laying hens has no significant effect on egg production and quality, but the yolk color gradually becomes lighter [31].

Given the lack of research on the use of an ABR–wheat mixture in the diets of laying ducks, this study aims to investigate the effects of varying levels of ABR–wheat mixture in the diet of laying ducks on laying performance, egg quality, yolk fatty acid profile, economic benefits, serum biochemistry and nutrient digestibility. Determining the appropriate level of ABR–wheat mixture to replace corn in equal amounts is beneficial for constructing a new feed formula structure and promoting the sustainable development of the livestock industry.

## 2. Materials and Methods

This study was approved by the Animal Care and Use Committee, Sichuan Agricultural University (Ethic Approval Code: SICAUAC202110-2; Chengdu, China).

### 2.1. Aged Brown Rice–Wheat Mixture

The ABR–wheat mixture used in the present experiment consisted of 85% ABR and 15% wheat, from Heilongjiang Grain Depot (Heilongjiang, China). The brown rice in the ABR–wheat mixture was stored for five years (2016–2021), and the wheat was stored for two years (2019–2021). The storage conditions comply with the requirements of GB/T 7415-2008 [32]. The storage quality of the ABR–wheat mixture and its separated aged brown rice and wheat is shown in Table 1. The content of aflatoxin B1, deoxynivalenol, and zearalenone in the ABR–wheat mixture used in the experiment were all below the legislative limits (GB 13078-2017) [33]. The acidity of fatty acids was determined by the national standard methods (GB/T 20570-2015) [34]. Determination of aflatoxin and zearalenone by national standard methods (NY/T 2071-2011) [35], and determination of deoxynivalenol by national standard methods (GB/T 30956-2014) [36].

### 2.2. Birds, Design, and Feeding Management

Birds were assigned to five treatments (0–50% ABR–wheat mixture; *n* = 120/treatment, 12/replicate). A total of 600 Jinding ducks (32 weeks old), with healthy and similar laying rates, were randomly divided into five treatment groups, with 10 replicates per group, and each replicate contained 12 ducks housed in cages. A single-factor experimental design was used with five treatments. The levels of ABR–wheat mixture in the diet were 0%, 12.5%, 25%, 37.5%, and 50%, to replace corn in the same amount of the control group diet. The basal diet was corn–soybean meal based on national standards (China) for nutrient requirements of egg-laying ducks (GB/T 41189-2021) [37]. All diets were formulated with isonitrogenous, isocaloric, and equal levels of digestible lysine, methionine, threonine and tryptophan (Table 2). All replicates were evenly distributed in the duck house. All experimental birds were raised in two-story step mesh cages, and each replicate comprised six consecutive cages with two birds per cage. The ducks were maintained in an environment with 16 h of light (from 04:00 a.m. to 08:00 p.m.) and were fed twice a day at 09:00 and 16:00. All of the diets were pelleted, and birds were given ad libitum access to drinking water and feed throughout the entire trial period. Eggs were collected daily at 9:30 AM, and the enclosure was cleaned regularly.

Following the feeding trial, a metabolic test was conducted to evaluate dietary nutrient digestibility using an exogenous indicator method. Titanium dioxide (TiO_2_) has stable chemical properties, is harmless to animals at recommended addition levels (generally 0.3–0.5%), has a high recovery rate (over 95%), and has high detection sensitivity, making it an ideal exogenous indicator [38,39]. In this experiment, 0.5% TiO_2_ was added into the feed as an exogenous indicator. Firstly, 80 healthy laying ducks with similar egg production rates were selected from the control group of experimental ducks. They were divided into five treatment groups, namely 0%, 12.5%, 25%, 37.5%, and 50% ABR–wheat mixture groups, with eight replicates per treatment and two egg ducks per replicate per cage. After a 5-day acclimatization period, excreta samples from each replicate were collected over the subsequent 3 days of the trial, followed by immediate preservation at −20 °C for further analyses. During the experimental periods, diets and water were provided ad libitum.

### 2.3. Laying Performance and Egg Quality Parameters

The total number of eggs produced, total egg weight, and the number of dead ducks were recorded daily. The provided and residual feed amounts of each replicate were documented weekly. The provided and residual feed amount of each replicate were recorded every week.

At 4, 8, and 12 weeks into the trial, three eggs were randomly selected from each replicate, and a total of 30 eggs were collected from each treatment group to assess egg quality, which included measurements of Haugh unit, albumen height, yolk color, eggshell strength, and eggshell thickness. Eggshell strength was measured using an eggshell strength tester (Model II, Robotmation Co., Ltd., Tokyo, Japan). Yolk color, albumen height and Haugh unit were evaluated with an egg quality automatic analyzer (EMT-5200, Robotmation Co., Ltd., Tokyo, Japan). The eggshell thickness gauge (Robotmation Co., Ltd., Tokyo, Japan) was employed to measure the thickness of the eggs in three distinct regions: the equatorial region, small end, and large end.

### 2.4. Performance Calculation

Duck-housed laying rate (%) = (total number of eggs/total number of housed ducks) × 100; Duck-day laying rate (%) = (total number of eggs/total number of ducks) × 100; The average egg weight (g) = total egg weight/number of eggs produced; Feed consumption (g/duck/day) = feed offered − feed leftovers; Feed-to-egg ratio = feed consumption/total egg weight; Egg mass (kg/bird) = total egg weight/number of housed ducks.

Total egg revenue (USD) = total egg weight (kg) × egg price (USD/kg); Total feed cost (USD) = total feed consumption (kg) × feed unit price (USD/kg); Total profit (USD/duck-housed) = (total egg revenue-total feed cost)/total number of housed ducks; Relative profit (USD/duck-housed) = Total profittreatment group − Total profitcontrol group.

### 2.5. Yolk Fatty Acid Profile Analysis

At the end of the experiment, three eggs were randomly collected from each replicate, and their yolks were mixed thoroughly. Analyses of the fatty acid composition were conducted according to the method described by Domaradzki et al. [40]. The egg yolk samples were freeze-dried at −80 °C to achieve a constant weight using a vacuum freeze dryer (FDU-2110, EYELA, Tokyo, Japan). The fatty acid profiles of the egg yolk samples were separated via gas chromatography (Agilent 8890A, Santa Clara, CA, USA). Lipids were extracted using a chloroform:methanol solution (2 mL:1 mL), which was vortexed for 2 min and then centrifuged at 1792 g for 10 min. Following the esterification process, fatty acid methyl esters (FAME) were produced from the supernatant using a methanol:sulfuric acid mixture (95 mL:5 mL). A sample volume of 1 microliter was injected into the injector, with an optimal flow rate of 1.0 mL/min for nitrogen as the carrier gas. The peaks corresponding to different fatty acids were identified by comparing their retention times with relevant standards. The content of various fatty acids was quantified based on the peak area and expressed as a percentage of the total fatty acids.

### 2.6. Serum Biochemistry Analysis

At the end of the 12th week of the trial, one laying duck from each replicate was randomly selected for blood collection. Blood was drawn from the jugular vein and divided into two collection tubes, where it was allowed to stand. Blood was centrifuged (3000 rpm, 10 min, 4 °C), and the upper serum layer was transferred into a sterile EP tube for use as the serum sample. This serum sample was then frozen at −20 °C. The total protein (TP), albumin (ALB), globulin (GLB), aspartate aminotransferase (AST), alanine aminotransferase (ALT), total cholesterol (TC), triglycerides (TG), low-density lipoprotein (LDL-C), and high-density lipoprotein (HDL-C) contents were determined using a fully automated biochemical analyzer (HATICHI 7180, Tokyo, Japan).

### 2.7. Nutrient Digestibility of Diet and Ingredients Chemical Analyses

The contents of dry matter (DM), ether extract (EE), and crude protein (CP) in ingredient, diet, and excreta samples, as well as the levels of crude fiber (CF) and ash in the ingredients, were measured following the guidelines outlined by AOAC International (2007) [41]. The gross energy (GE) of the ingredients, diet, and excreta samples was determined using an automatic bomb calorimeter (Parr 6400, Parr Instruments, Moline, IL, USA). The methodology for determining the fatty acid profile of the ingredients mirrored that used for the fatty acid profile of egg yolk. TiO_2_ concentrations in the diets and excreta were analyzed according to the procedure described by Short et al. [39]. The data regarding the composition of the diets and excreta were utilized to calculate the digestibility coefficients for DM, CP, GE, and EE.

Digestibility of nutrient (%) = [1 − (TiO_2 diet_/TiO_2 excreta_) × (Nutrient_excreta_/Nutrient_diet_)] × 100

### 2.8. Statistical Analysis

Data were analyzed using SPSS version 25.0, employing one-way analysis of variance (ANOVA, New Providence, NJ, USA) for the analysis, along with linear and quadratic regression analyses, and Duncan’s method for multiple comparisons. Results are presented as means with their corresponding standard errors. Statistical significance was determined at *p* < 0.05.

## 3. Results

### 3.1. Ingredients Chemical Analyses

The nutrient composition of the corn and ABR–wheat mixture used in the experiments is detailed in Table 3. On an air-dry basis, the GE, CP, and EE of the ABR–wheat mixture were 15.49 MJ/kg, 7.84%, and 1.9%, respectively, reflecting reductions of 0.32 MJ/kg, 0.13%, and 1.06% in comparison to corn; the CF and ash levels of the ABR–wheat mixture were 3.45% and 1.57%, respectively, reflecting increases of 1.25% and 0.4% in comparison to corn. The relative content of palmitic, palmitoleic, stearic, and oleic in the ABR–wheat mixture was higher than that in corn, while the relative content of linoleic and linolenic was lower than that in corn.

### 3.2. Laying Performance and Egg Quality

Table 4 shows that replacing corn with different levels of ABR–wheat mixture did not significantly affect duck-housed laying rate, duck-day laying rate, average egg weight, feed consumption, feed-to-egg mass ratio, and egg mass of laying ducks (*p* > 0.05). From a numerical perspective, the 37.5% group had a higher duck-housed laying rate and egg mass compared to other treatment groups, while the 50% group had the lowest. Table 5 demonstrates that different treatments had no significant effect on the eggshell strength and thickness of duck eggs at weeks 4, 8, and 12 (*p* > 0.05). As the level of ABR–wheat mixture increased, yolk color showed a linear decrease at weeks 4, 8, and 12 (*p* < 0.05); the albumen height and Haugh unit showed a quadratic increase (*p* < 0.05) at weeks 4 and 8, with the highest values observed in the 25% and 37.5% groups. However, there was no significant difference between the treatments at week 12 (*p* > 0.05).

### 3.3. Yolk Fatty Acid Profile

As demonstrated in Table 6, the primary fatty acids in duck egg yolk of each treatment are palmitic (C16:0), stearic (C18:0), oleic (C18:1), and linoleic (C18:2). As the level of ABR–wheat mixture increased, the relative content of palmitoleic (C16:1), oleic (C18:1), and total MUFA increased linearly (*p* < 0.05), while the relative content of tricosanoic (C23:0), linoleic (C18:2), linolenic (C18:3), eicosadienoic (C20:2), docosahexaenoic (C22:6), ∑ω-3 PUFA, ∑ω-6 PUFA and ∑ω-6/∑ω-3, as well as total PUFA, decreased linearly (*p* < 0.05).

### 3.4. Economic Benefits

Based on the market price at the time of the experiment, the unit price of corn was 0.48 USD/kg, the unit price of ABR–wheat mixture was 0.47 USD/kg, and the selling price of duck eggs was 1.78 USD/kg. Net profit peaked at 0.10 USD/duck (37.5% substitution). As shown in Table 7, as the level of ABR–wheat mixture increased, the unit price of feed gradually decreased. Compared with the 0% group, the relative benefits of each duck-housed in the 12.5%, 25%, 37.5%, and 50% groups were 0.04 USD, 0.01 USD, 0.10 USD, and −0.04 USD, respectively.

### 3.5. Serum Biochemistry

As shown in Table 8, different levels of ABR–wheat mixture in the diet to replace equal amounts of corn had no significant effect on the serum biochemical indicators (*p* > 0.05).

### 3.6. Nutrient Digestibility of Diet

As demonstrated in Table 9, as the level of ABR–wheat mixture increased, the digestibility of GE, EE and CP of the diet, as well as AME of the diet, decreased linearly (*p* < 0.05). The CP digestibility of the 50% group was significantly lower than that of the other treatment groups (*p* < 0.05), while there was no significant difference between the 0% to 37.5% treatment groups (*p* > 0.05).

## 4. Discussion

Brown rice and wheat are common preferred energy feed ingredients to replace corn, but the content and quality of the main nutrients in grains undergo certain changes during storage [42]. Fatty acid value is an important parameter for evaluating the quality of grain storage, and the longer the storage time, the higher the fatty acid value [43].

Elevated fatty acid values (59.0 mgKOH/100 g) indicated lipid oxidation in ABR–wheat. In the mixture, wheat had a fatty acid value of 22.7 mgKOH/100 g, and brown rice had a value of 62.9 mgKOH/100 g. According to Chinese standards GB/T 20569-2006 [44] and GB/T 1355-2021 [45], the brown rice was aged, and the wheat was normal. In addition, during the storage process, grains are prone to infection with fungal toxins, leading to mold and contamination of livestock feed [46]. The ABR–wheat mixture had 5.23 µg/kg of aflatoxin B1, 0.6 mg/kg of deoxynivalenol, and 0 µg/kg of zearalenone. These amounts were all less than the Chinese standard GB 13078-2017. The ABR–wheat mixture had undergone a certain degree of oxidative rancidity, which may affect the application effect of laying ducks. The content of mycotoxins was below the limit and could be safely used for the preparation of laying duck feed.

The issue of reducing and replacing corn in animal feed mainly focuses on one of brown rice or wheat, while there are few reports on the application of aged brown rice–wheat mixture in livestock and poultry. In this experiment, different levels of ABR–wheat mixture in the diet had no significant effect on the production performance of laying ducks. A similar result was obtained by He et al. [5], who reported that the complete replacement of corn with brown rice stored for 6 years had no significant effect on the growth performance and meat quality of weaned piglets. Meanwhile, He et al. [27] also found that replacing corn with brown rice stored for 6 years or 4 years in the diet would not have adverse effects on the growth and slaughter performance of broilers. But Li [47] reported that feeding a three-year-stored brown rice diet would reduce feed conversion rate and increase feed-to-weight ratio. In addition, replacing corn with 30% wheat did not affect the egg production rate, egg weight, feed intake and feed conversion rate of laying hens from 27 to 43 week of age [48]. Wang et al. [49] also found that the laying rate of laying hens significantly decreased when normal corn was 100% replaced by ageing corn stored for 4 years. Improper storage of grains can easily lead to mold growth and adding them to feed can cause contamination with mycotoxins [50]. The application effect of stored grain in livestock and poultry feed is inconsistent, which may be related to the storage time, storage conditions, and quality of the stored grain. Therefore, in practical applications, the nutritional value of stored grain should be comprehensively evaluated based on factors such as nutritional composition, safety indicators, and storage quality.

The yolk color, albumen height, and Haugh unit are important indicators for evaluating egg quality. The yolk color is associated with carotenoids [51]. However, poultry cannot synthesize these carotenoids and must obtain them from their diet [52]. Sittiya et al. [14] reported that yolk color significantly decreased with increasing levels of paddy rice (50% or more). In this study, it was also found that, as the replacement level of the ABR–wheat mixture increased, the yolk color linearly decreased. The reason may be that corn is rich in carotene and lutein, and it is the main source of pigments in animal diets [53], while brown rice and wheat contain almost no carotene [22], resulting in reduced deposition of yolk pigments and lighter yolk colors. Therefore, in the practical application of an ABR–wheat mixture, it is possible to consider adding an appropriate amount of pigments to the diet to improve the yolk color [31]. Albumen height and Haugh unit are important indicators for evaluating egg white quality and are also the most commonly used indicators to reflect freshness. The larger the Haugh unit, the thicker the protein, indicating better protein quality and freshness of duck eggs. Zhou et al. [30] reported that dietary ageing corn linearly decreases the albumen height. An et al. [11] found that adding 0–15% of brown rice stored for 3 years to the diet had no significant effect on the Haugh unit of eggs. However, this study found that, in weeks 4 and 8 of the experiment, albumen height and Haugh unit increased, with the 25.5% and 50% groups being significantly higher than the control group. The inconsistency with previous research findings may be attributed to the complementary nature of amino acids when brown rice and wheat are combined [19]. The ABR–wheat mixture may improve protein height by optimizing amino acid utilization and promoting the deposition of viscoelastic proteins in egg whites.

The fatty acid composition in the egg yolk is closely related to the fatty acid composition in the diet [54,55], which is also the main theoretical basis for producing poultry eggs containing PUFA in the market. By adding ingredients rich in PUFA, such as fish oil and flaxseed to the diet, the content of PUFA in poultry eggs can be increased [54,55]. Corn or an ABR–wheat mixture is the main component of the diet, so the profile of fatty acid in corn or the ABR–wheat mixture can affect the profile of fatty acid in egg yolk. This study found that an ABR–wheat mixture can change the fatty acid profile of duck egg yolks. As the level of ABR–wheat mixture increased, the relative content of MUFA in egg yolks increased linearly, especially C16:1 and C18:1, while the relative content of PUFA decreased linearly, especially C18:2, C18:3, and C22:6. Similar to our study, Zhou et al. [56] found that replacing normal corn with ageing corn can lead to a decrease in PUFA in egg yolks. There is a positive correlation between tissue fatty acid composition and dietary fatty acid composition [57,58]. PUFA are easily oxidized during storage [59]. In the analysis of ingredients composition, the main fatty acids in the ABR–wheat mixture, such as C18:2 and C18:3, were significantly lower than those in corn in this experiment, while C16:1 and C18:1 were significantly higher than those in corn. The lower content of PUFA and higher content of MUFA in ABR–wheat mixture correspond to the lower content of PUFA and higher content of MUFA in egg yolk. ω-6 PUFA and ω-3 PUFA are important components of cell membranes and precursors of eicosanolic acids [60]. They have a positive effect on the cardiovascular system by reducing cholesterol levels, triglyceride synthesis, inhibiting platelet aggregation, and lowering blood pressure [61]. And 22:6 ω-3 is essential for brain and visual development [62]. ω-6 PUFA and ω-3 PUFA are important for human health and can alleviate many diseases [63]. However, the human body is incapable of synthesizing them directly and must get them through dietary sources. In this study, the ABR–wheat mixture significantly reduced the ω-3 PUFA and ω-6 PUFA content in the egg yolks, thereby reducing the nutritional value of duck eggs.

In this study, as the replacement level of the ABR–wheat mixture increased, the feed unit price gradually decreased. The 12.5%, 25%, 37.5%, and 50% ABR–wheat mixture groups replaced the same amount of corn in the feed, respectively. The profit per housed duck in the 12.5% group, 25% group, and 37.5% group was higher than that in the control group, while the profit per housed duck in the 50% group was lower than that in the control group. The control group incurred a feed cost of USD 0.514/kg, while the 37.5% substitution group demonstrated a reduced cost of USD 0.500/kg, representing a cost reduction of USD 13.38 per tonne. At the same time, the 37.5% substitution group achieved superior profitability, with a higher profit per housed duck compared to the other treatment groups throughout the 12-week experimental period. Therefore, although the ABR–wheat mixture lacks suitability for human consumption, the reasonable use of the ABR–wheat mixture can bring economic benefits and replace some of the corn in the diet of laying ducks, which is of great significance for the feed industry in the context of corn shortage. However, in commercial feed production, ingredient prices exhibit significant market volatility, necessitating strategic selection of optimal substitution ratios in formulas design based on prevailing cost.

Serum biochemical indicators can reflect the health and nutritional status of animals. Mu et al. [29] found that ageing corn had no significant effect on the serum biochemical parameters of laying hens. Compared with newly harvested brown rice, stored brown rice did not significantly influence serum biochemical indicators in broiler chickens [27]. This study revealed that the ABR–wheat mixture exerted no significant influence on the serum biochemical indicators of laying ducks, indicating that its application did not cause abnormalities in energy metabolism, glucose metabolism, and protein metabolism in these birds. However, the ABR–wheat mixture may influence serum lipid metabolism. In this experiment, the TG content in the serum showed a curve of first increasing and then decreasing. The 50% ABR–wheat mixture group significantly reduced the TG content in the serum. Mu et al. [29] observed a comparable outcome, indicating that ageing corn reduced the serum TG content of laying hens. Yue et al. [64] also found that adding dietary oxidized oil into the diets of laying hens reduced serum TG content. Resistant starch in aged grains may promote SCFA production [65,66,67]. Reduced serum TG (50% group) likely reflects SCFA-mediated AMPK activation, which inhibits acetyl-CoA carboxylase and fatty acid synthase, thereby diminishing hepatic fat production [68]. On the other hand, SCFA upregulates the activation of peroxisome proliferator-activated receptor alpha, accelerating the beta oxidation of fatty acids [69].

Nutrient digestibility is an important indicator for measuring the health status of animals and the nutritional value of feed [70]. Wang et al. [49] showed that replacing 50% and 100% normal corn with ageing corn significantly reduced the activity of trypsin in the foregut chyme of laying hens, suggesting that ageing corn can affect the digestion of protein in laying hens. Storing corn separately for 1, 2, 3, 4, and 5 years, the results indicated that, as the storage time increased, there was a linear decrease in the crude protein digestibility in the corn of pigs [71]. The results of this study also showed that the apparent digestibility of crude protein in the diet of laying ducks was negatively correlated with the level of ABR–wheat mixture replacing corn, and the 50% group was significantly lower than that of the other treatment groups. This may be related to the antinutritional factors contained in brown rice and wheat. Brown rice contains pepsin inhibitors, while wheat contains non-starch polysaccharides, both of which can affect protein digestion and utilization [20,21]. When replaced at a level of 0–37.5%, antinutritional factors may not reach a significant level that interferes with digestion. However, complete substitution at a 50% level may significantly inhibit protease activity, thereby reducing the digestibility of CP. Liu and Huang [72] studied mice fed a 15% oxidized soybean oil diet and a 15% fresh soybean oil diet, and found that the fat utilization rate of the oxidized soybean oil diet decreased by 4.96%. Mu [73] found that completely replacing normal corn with ageing corn stored for 4 years significantly reduced the utilization rate of ether extract in the diet of laying hens. Similar findings were also made in this experiment, where the digestibility of EE in the diet decreased linearly. The activity of lipase is influenced by the variation in fatty acids present in the diet [74], with bile salt-activated lipase showing a higher affinity for polyunsaturated fatty acids [75]. In this study, the PUFA content of the ABR–wheat mixture was notably lower compared to corn, resulting in a gradual decline in the digestibility of EE in the diet. Additionally, the ABR–wheat mixture was found to induce oxidative stress in the body, which can diminish cellular metabolic capacity, as well as hinder and modify cell function, ultimately leading to a reduction in nutrient absorption. In our study, the AME in the diet decreased linearly as the level of ABR–wheat mixture in the diet increased. This may be due to the metabolic energy of brown rice and wheat in the experimental feed formula being based on theoretical values for laying ducks, while the effective energy of grains may diminish during storage [71]. This may explain why, in this experiment, feed consumption increased numerically with the increase in ABR–wheat mixture level, while production performance did not improve. The laying rate and total egg mass of the 50% group decreased numerically, which may be attributable to a decline in the diet’s nutrition utilization efficiency.

Under the conditions of this experiment, the ABR–wheat mixture has certain cost advantages and resource utilization value as feed in duck farming, but there may also be limitations such as short-term effects and lack of mycotoxin mitigation strategies. In the actual promotion process, there are also some limitations and challenges, such as lighter yolk color and reduced yolk nutritional value, and the risk of feed contamination by mycotoxins. Moreover, the ABR–wheat mixture contains antinutritional factors that affect animal digestion and absorption. It is necessary to add exogenous enzymes to the feed to remove these antinutritional factors. At the same time, the ABR–wheat mixture may have significant differences in nutritional content due to different storage times and conditions, which poses challenges for feed formulations. To address these issues, we may need to further research and develop fermentation or enzymatic hydrolysis processes to enhance its nutritional value, safety, and stability.

## 5. Conclusions

The inclusion of different levels of ABR–wheat mixture in the diet as a replacement for equivalent amounts of corn resulted in lighter yolk color and altered fatty acid composition, reducing PUFA content while increasing MUFA levels. A high-level ABR–wheat mixture (50%) would significantly reduce the TG content in serum and nutrient digestibility of the diet, and also have a certain negative impact on laying rate and egg mass numerically. Based on laying performance and economic benefits, a 37.5% ABR–wheat mixture is recommended to replace an equal amount of corn in the diet of 32- to 43-week-old laying ducks.

## Figures and Tables

**Table 1:** Storage quality of ABR–wheat mixture and its separated aged brown rice and wheat.

Items	ABR–Wheat Mixture	ABR	Wheat
Acidity of fatty acids/(mgKOH/100 g)	59.00 ± 1.08	62.90 ± 1.21	22.70 ± 0.31
Mycotoxin			
Aflatxin B_1_/(μg/kg)	5.23 ± 0.05	2.80 ± 0.03	-
Deoxynivalenol/(mg/kg)	0.60 ± 0.03	0.50 ± 0.02	1.10 ± 0.04

Note: “-” Indicates not detected.

**Table 2:** Composition and nutrient levels of the experimental diets (%, air-dry basis).

Items	ABR–Wheat Mixture Levels, %				
	0	12.5	25	37.5	50
Ingredients					
Corn	50.00	37.50	25.00	12.50	0.00
ABR–wheat mixture ^1^	0.00	12.50	25.00	37.50	50.00
Soybean meal (CP43%)	25.70	25.78	25.85	25.93	26.00
Wheat bran	11.00	10.60	10.20	9.80	9.40
Wheat flour	1.25	1.25	1.25	1.25	1.25
Soybean oil	1.00	0.83	0.65	0.48	0.30
Limestone	8.42	8.43	8.44	8.44	8.45
CaHPO_4_	0.62	0.60	0.59	0.57	0.55
*L*-Lysine-HCL (98.5%)	0.05	0.04	0.03	0.01	0.00
*DL*-Methionine (99%)	0.14	0.14	0.14	0.14	0.14
*L*-Threonine (98.5%)	0.01	0.01	0.01	0.02	0.02
*L*-Tryptophan (98%)	0.02	0.02	0.01	0.01	0.01
NaCl	0.55	0.55	0.55	0.55	0.55
Chline chloride (50%)	0.12	0.12	0.12	0.12	0.12
Mineral premix ^2^	0.30	0.30	0.30	0.30	0.30
Vitamin premix ^3^	0.02	0.02	0.02	0.02	0.02
Other	0.80	1.32	1.85	2.37	2.89
Total	100.00	100.00	100.00	100.00	100.00
Calculated value of nutrition level (%)					
AME (MJ/kg)	10.69	10.69	10.69	10.69	10.69
Crude protein	17.01	17.01	17.01	17.01	17.01
Calcium	3.50	3.50	3.50	3.50	3.50
Nonphytate phosphorus	0.25	0.25	0.25	0.25	0.25
Digestible Lysine	0.81	0.81	0.81	0.81	0.81
Digestible Methionine	0.37	0.37	0.37	0.37	0.37
Digestible Threonine	0.56	0.56	0.56	0.56	0.56
Digestible Tryptophan	0.20	0.20	0.20	0.20	0.20

^1^ When calculating the nutritional level of feed formula, the AME of aged brown rice–wheat mixture referred to the nutrient requirements of egg ducks (GB/T 41189-2021) for brown rice and wheat, calculated as aged brown rice–wheat mixture = 85% brown rice + 15% wheat; The crude protein content of ABR–wheat mixture was the measured value. ^2^ The mineral premix provided the following per kg of diets: Cu (CuSO_4_·5H_2_O), 20 mg; Fe (FeSO_4_·H_2_O), 50 mg; Mn (MnSO_4_·H_2_O), 82 mg; Zn (ZnSO_4_·H_2_O), 65 mg; I (KI), 0.4 mg; Se (Na_2_SeO_3_), 0.25 mg. ^3^ The vitamin premix provided the following per kg of diets: VA 8000 IU; VD_3_ 2000 IU; VE 20 IU; VK_3_ 3 mg; VB_1_ 2 mg; VB_2_ 6.4 mg; VB_6_ 4 mg; VB_12_ 0.02 mg; niacin acid 40 mg; pantothenic 12 mg; biotin 0.11 mg; folic acid 1.0 mg.

**Table 3:** Analyzed nutrient composition of ingredients used in the experiment (%, air-dry basis).

Items	Corn	ABR–Wheat Mixture
Dry matter	86.32	86.29
Gross energy (MJ/Kg)	15.71	15.49
Crude protein	7.97	7.84
Crude fat	2.96	1.90
Crude fiber	2.27	3.45
Ash	1.17	1.57
Fatty acid profile, %		
Palmitic (C16: 0)	11.84	18.36
Palmitoleic(C16: 1)	0.09	0.20
Stearic (C18: 0)	2.92	3.11
Oleic (C18: 1)	26.50	35.73
Linoleic (C18: 2)	56.79	35.93
Linolenic (C18: 3)	1.68	1.50

**Table 4:** Effects of ABR–wheat mixture on laying performance of laying ducks.

Items	ABR–Wheat Mixture Levels, %					SEM	*p*-Value		
	0	12.5	25	37.5	50		Anova	Linear	Quadratic
Duck-housed laying rate (%)	86.65	85.82	86.35	87.00	85.15	2.07	0.911	0.687	0.866
Duck-day laying rate (%)	87.23	86.87	87.06	87.14	85.68	2.07	0.942	0.532	0.751
Average egg weight (g)	73.15	73.81	73.00	73.44	73.02	0.50	0.442	0.572	0.676
Feed consumption/(g/duck/day)	181.74	183.37	185.53	185.23	184.97	3.18	0.735	0.236	0.376
Feed-to-egg ratio/(g:g)	2.88	2.89	2.94	2.92	3.00	0.08	0.545	0.110	0.270
Weekly egg mass/(kg/bird)	5.32	5.31	5.28	5.36	5.22	0.14	0.878	0.619	0.792

**Table 5:** Effects of ABR–wheat mixture on egg quality of laying ducks.

Items	ABR–Wheat Mixture Levels, %					SEM	*p*-Value		
	0	12.5	25	37.5	50		Anova	Linear	Quadratic
Albumen height (mm)									
4 week	6.94 ^c^	7.18 ^bc^	8.30 ^a^	8.07 ^ab^	7.90 ^abc^	0.51	0.033	0.058	0.059
8 week	6.86 ^b^	7.82 ^ab^	8.09 ^a^	8.02 ^a^	7.46 ^ab^	0.49	0.079	0.593	0.016
12 week	7.85	7.84	7.75	7.74	7.76	0.41	0.998	0.371	0.671
Haugh unit									
4 week	78.38 ^c^	79.72 ^bc^	86.56 ^a^	84.88 ^ab^	83.13 ^abc^	2.88	0.026	0.090	0.040
8 week	77.06 ^b^	83.03 ^a^	85.95 ^a^	84.60 ^a^	81.96 ^ab^	2.83	0.024	0.317	0.018
12 week	83.91	83.88	83.48	84.06	83.78	2.37	0.999	0.471	0.751
Yolk color									
4 week	13.55 ^a^	13.05 ^b^	12.90 ^b^	12.75 ^b^	12.85 ^b^	0.21	0.001	0.005	<0.001
8 week	13.37 ^a^	12.88 ^b^	12.78 ^bc^	12.43 ^cd^	12.31 ^d^	0.19	<0.001	<0.001	<0.001
12 week	12.59 ^a^	12.35 ^ab^	12.12 ^bc^	12.08 ^bc^	11.71 ^c^	0.22	0.002	0.010	0.032
Eggshell strength (kg/cm^2^)									
4 week	4.85	4.70	4.81	4.75	4.81	0.08	0.394	0.699	0.744
8 week	4.61	4.68	4.52	4.38	4.54	0.13	0.196	0.268	0.544
12 week	4.51	4.59	4.50	4.48	4.61	0.14	0.849	0.799	0.599
Eggshell thickness (mm)									
4 week	0.37	0.37	0.37	0.37	0.37	0.00	0.975	0.982	0.748
8 week	0.37	0.37	0.36	0.36	0.37	0.00	0.302	0.360	0.612
12 week	0.35	0.36	0.36	0.36	0.36	0.00	0.177	0.636	0.356

Different lowercase letters of peer shoulder notes indicate significant differences (*p* < 0.05), and no letters or identical letters indicate no significant difference (*p* > 0.05).

**Table 6:** Effects of ABR–wheat mixture on yolk fatty acid profile of laying ducks (%).

Items	ABR–Wheat Mixture Levels, %					SEM	*p*-Value		
	0	12.5	25	37.5	50		Anova	Linear	Quadratic
Myristic (C14:0)	0.42	0.42	0.42	0.43	0.43	0.02	0.992	0.797	0.896
Palmitic (C16:0)	25.05	24.95	24.98	25.02	25.11	0.38	0.995	0.656	0.774
Stearic (C18:0)	6.22	6.28	5.92	6.22	5.97	0.13	0.064	0.118	0.298
Tricosanoic (C23:0)	2.46 ^a^	2.40 ^a^	2.33 ^ab^	2.20 ^bc^	2.11 ^c^	0.08	<0.001	<0.001	<0.001
Palmitoleic (C16:1)	2.41 ^c^	2.75 ^b^	2.64 ^bc^	2.79 ^b^	3.18 ^a^	0.12	<0.001	<0.001	<0.001
Oleic (C18:1)	49.09 ^c^	50.25 ^c^	52.39 ^b^	52.71 ^ab^	53.80 ^a^	0.64	<0.001	<0.001	<0.001
Linoleic (C18:2)	11.29 ^a^	10.02 ^b^	8.63 ^c^	7.92 ^d^	6.96 ^e^	0.31	<0.001	<0.001	<0.001
Linolenic (C18:3)	0.85 ^a^	0.77 ^b^	0.67 ^c^	0.63 ^c^	0.57 ^d^	0.02	<0.001	<0.001	<0.001
Eicosadienoic (C20:2)	0.31 ^a^	0.27 ^b^	0.25 ^c^	0.22 ^d^	0.20 ^d^	0.01	<0.001	<0.001	<0.001
Eicosatrienoic (C20:3)	0.42	0.44	0.40	0.40	0.41	0.02	0.402	0.309	0.599
Docosahexaenoic (C22:6)	0.79 ^a^	0.75 ^ab^	0.72 ^bc^	0.69 ^bc^	0.67 ^c^	0.02	<0.001	<0.001	<0.001
∑SFA	34.62	34.65	34.08	34.31	34.03	0.31	0.188	0.217	0.443
∑UFA	65.38	65.35	65.92	65.69	65.97	0.31	0.188	0.103	0.254
∑MUFA	51.70 ^d^	53.21 ^c^	55.24 ^b^	55.79 ^b^	57.14 ^a^	0.55	<0.001	<0.001	<0.001
∑PUFA	13.69 ^a^	12.30 ^b^	10.68 ^c^	9.91 ^d^	8.83 ^e^	0.35	<0.001	<0.001	<0.001
∑ω-3 PUFA	1.49 ^a^	1.38 ^b^	1.27 ^c^	1.23 ^c^	1.14 ^d^	0.03	<0.001	<0.001	<0.001
∑ω-6 PUFA	11.88 ^a^	10.63 ^b^	9.15 ^c^	8.45 ^d^	7.49 ^e^	0.33	<0.001	<0.001	<0.001
∑ω-6/∑ ω-3	7.95 ^a^	7.69 ^a^	7.18 ^b^	6.88 ^bc^	6.62 ^c^	0.21	<0.001	<0.001	<0.001

Different lowercase letters of peer shoulder notes indicate significant differences (*p* < 0.05), and no letters or identical letters indicate no significant difference (*p* > 0.05).

**Table 7:** Effects of ABR–wheat mixture on economic benefits of laying ducks (%).

Items	ABR–Wheat Mixture Levels, %				
	0	12.5	25	37.5	50
Feed unit price (USD/kg)	0.514	0.509	0.505	0.500	0.496
Total egg weight (kg)	94.87	94.76	94.29	95.64	93.09
Total feed consumption (kg)	270.6	271.3	273.1	277.1	275.4
Total egg revenue (USD)	1138.4	1137.2	1131.5	1147.7	1117.0
Total feed cost (USD)	934.8	929.0	927.1	932.5	918.7
Total profit (USD)	203.6	208.2	204.4	215.2	198.3
Total profit (USD/duck-housed)	1.70	1.73	1.70	1.79	1.65
Relative profit (USD/duck-housed)	0.00	0.04	0.01	0.10	−0.04

**Table 8:** Effects of ABR–wheat mixture on serum biochemical indicators of laying ducks (%).

Items	ABR–Wheat Mixture Levels, %					SEM	*p*-Value		
	0	12.5	25	37.5	50		Anova	Linear	Quadratic
Total protein (g/L)	48.40	51.57	53.07	48.79	51.98	4.16	0.540	0.324	0.207
Albumin (g/L)	23.90	25.19	30.70	23.02	27.27	4.06	0.342	0.613	0.689
Globulin (g/L)	22.84	23.40	22.37	24.10	22.80	2.21	0.947	0.900	0.983
Alanine aminotransferase (U/L)	19.13	26.68	24.84	21.01	20.72	3.64	0.211	0.778	0.179
Aspartate aminotransferase (U/L)	16.19	20.86	20.15	22.44	15.01	4.43	0.430	0.994	0.220
Total cholesterol/(mmol/L)	2.30	2.61	2.34	2.35	2.44	0.31	0.879	0.994	0.977
Triglycerides/(mmol/L)	5.47 ^b^	6.58 ^a^	7.07 ^a^	5.20 ^b^	4.29 ^c^	0.94	0.039	0.080	0.012
Low-density lipoproteincontents/(mmol/L)	0.95	1.11	0.96	0.98	0.80	0.14	0.313	0.164	0.159
High-density lipoproteincontents/(mmol/L)	0.96	0.94	0.96	0.96	0.93	0.13	0.999	0.865	0.978

Different lowercase letters of peer shoulder notes indicate significant differences (*p* < 0.05), and no letters or identical letters indicate no significant difference (*p* > 0.05).

**Table 9:** Effects of ABR–wheat mixture on nutrient digestibility of diet in laying ducks.

Items	ABR–Wheat Mixture Levels, %					SEM	*p*-Value		
	0	12.5	25	37.5	50		Anova	Linear	Quadratic
GE	70.28 ^ab^	70.20 ^ab^	71.09 ^a^	69.33 ^b^	68.95 ^b^	0.72	0.042	0.042	0.032
DM	59.44 ^b^	59.49 ^b^	61.75 ^a^	59.49 ^b^	59.35 ^b^	0.79	0.013	0.948	0.282
CP	56.69 ^a^	55.89 ^a^	56.30 ^a^	53.19 ^a^	44.75 ^b^	3.70	0.016	<0.001	<0.001
EE	79.57 ^a^	78.54 ^a^	68.94 ^b^	70.39 ^b^	68.17 ^b^	3.22	0.002	0.003	0.004
AME (MJ/Kg)	10.38 ^a^	10.25 ^a^	10.30 ^a^	10.03 ^b^	9.98 ^b^	0.11	0.002	<0.001	<0.001

Different lowercase letters of peer shoulder notes indicate significant differences (*p* < 0.05), and no letters or identical letters indicate no significant difference (*p* > 0.05).

## Data Availability

Data are contained within the article.
